# Curcumin ameliorates hyperuricemia and gout‐induced damage via modulating the ROS‐dependent NEK7‐NLRP3 inflammasome activation

**DOI:** 10.1002/smo.20250002

**Published:** 2025-06-11

**Authors:** Zhengtian Zhao, Xiaowei Tong, Jing Sun, Jiaqi Lu, Guangyu Zhang, Qing Wang, Li Yang

**Affiliations:** ^1^ State Key Laboratory of Fine Chemicals Department of Pharmaceutical Engineering School of Chemical Engineering Dalian University of Technology Dalian China; ^2^ Ningbo Institute of Dalian University of Technology Ningbo China

**Keywords:** gout, hyperuricemia, NLRP3 inflammasome, pyroptosis

## Abstract

Curcumin, a bioactive compound extracted from *Curcuma longa.* L., demonstrates significant therapeutic potential in inflammatory diseases. This study aims to explore the effects of curcumin on hyperuricemia with acute gout and associated renal dysfunction in a mouse model. The results show that curcumin treatment alleviates ankle joint swelling, reduces inflammatory cytokines IL‐1*β* and TNF‐*α*, and lowers serum uric acid concentrations. High‐dose curcumin notably inhibits xanthine oxidase (XOD) activity, a key enzyme in uric acid production, while it enhances the renal expression of the urate transporter ABCG2, thereby promoting uric acid excretion. Furthermore, curcumin effectively mitigates renal injury as evidenced by reduced serum creatinineand blood urea nitrogen levels and suppresses renal inflammation. At the molecular level, curcumin exerts potent antioxidant effects by lowering reactive oxygen species (ROS) levels in both cultured HK‐2 human renal tubular epithelial cells and RAW264.7 mouse macrophages. The curcumin‐mediated effects are associated with the disruption of NEK7‐NLRP3 complex formation, leading to the suppression of the ROS/NEK7‐NLRP3 inflammasome pathway. This, in turn, inhibits pyroptosis and the subsequent release of mature IL‐1*β*. These findings suggest that curcumin not only reduces uric acid production but also modulates inflammation through ROS‐scavenging properties and its ability to inhibit the NLRP3 inflammasome.

## Graphical abstract

Curcumin inhibits the ROS/NEK7‐NLRP3 inflammasome pathway by reducing reactive oxygen species (ROS) levels and disrupting the association of the NEK7‐NLRP3 complex. These combined effects of curcumin result in suppression of the GSDMD‐N pyroptotic pore formation and subsequent reduction of IL‐1*β* release.

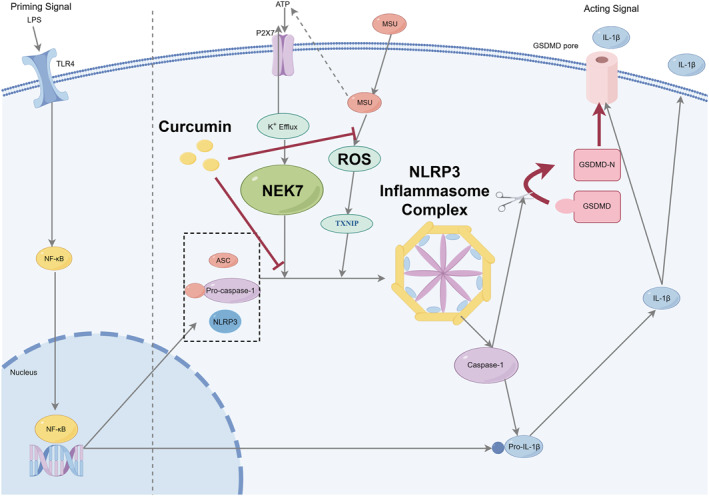

## 1 INTRODUCTION

Hyperuricemia refers to a condition defined by abnormally elevated levels of uric acid in the blood, primarily caused by excessive uric acid production^[1]^ and insufficient renal and intestinal excretion of uric acid.[^2^, ^3^] Sustained hyperuricemia induces chronic inflammation and progressive renal damage,[^4^, ^5^] including glomerular arteriosclerosis and renal tubular injury.^[6]^ This renal dysfunction further compromises uric acid clearance and promotes monosodium urate (MSU) crystal deposition in periarticular tissues.^[7]^ These deposits provoke the release of inflammatory mediators, triggering acute inflammatory responses[^8^, ^9^] and progressive articular damage, which ultimately manifest as gout.[^10^, ^11^]

Both the over‐synthesis of endogenous uric acid and the deposition of MSU crystals trigger the generation and release of reactive oxygen species (ROS),[^12^, ^13^] which activate oxidative stress responses and inflammatory cascades. These events converge on the NLRP3 inflammasome complex and further trigger the NLRP3 pathway. NLRP3 inflammasome assembly involves the interactions of NLRP3 with NEK7 and the recruitment of pro‐caspase‐1 through the ASC protein's CARD domain.^[14]^ The assembled complex leads to caspase‐1 activation, which cleaves the gasdermin D (GSDMD) to create a pore‐forming fragment (N‐GSDMD). Simultaneously, activated caspase‐1 processes pro‐IL‐1*β* into Cleaved‐IL‐1*β*, which can be secreted through the N‐GSDMD pore, further amplifies inflammation and induces pyroptosis.^[15]^ Studies indicate that urate salts can activate the NLRP3 inflammasome linking hyperuricemia to pyroptosis.^[16]^

Current treatment strategies for hyperuricemia and gout management focus on reducing uric acid levels and controlling inflammation. However, the clinical utility of urate‐lowering agents (e.g., allopurinol,^[17]^ benzbromarone^[18]^) or anti‐inflammatory therapies (colchicine,^[19]^ NSAIDs, corticosteroids) is constrained by adverse effects including post‐crystal dissolution inflammatory flares, hepatotoxicity and drug accumulation in renal impairment.^[20]^ These limitations highlight the need for safer therapeutic alternatives.

As a natural component and small molecular compound extracted from the rhizome of *Curcuma longa.* L.,[^21^, ^22^] curcumin has garnered attention for its broad pharmacological activities in both preclinical experiments and clinical trials.[^23^, ^24^] Experimental studies suggest that curcumin possesses a variety of biological activities, including antioxidant, anti‐inflammatory, ferroptosis inhibition, and ROS suppression.[^25^, ^26^, ^27^]

In this study, we employed a hyperuricemia with gouty arthritis mouse model to evaluate the curcumin's therapeutic efficay. The underlying mechanism was further elucidated that curcumin exerts its effects through the regulation of urate transport proteins and the modulation of the ROS/NEK7‐NLRP3 signaling pathway with disturbing interaction of NEK7 and NLRP3.

## 2 MATERIALS AND METHODS

### 2.1 Chemicals and reagents

Curcumin (cat. no. B20614), potassium oxonate (cat. no. S17112), allopurinol (cat. no. S45825), colchicine (cat. no. S18047) were purchased from Yuanye (Shanghai, China). Monosodium urate (MSU, cat. no. U886060) was purchased from Macklin (Shanghai, China). ELISA kits for IL‐1*β* (cat. no. U886060) and TNF‐*α* (cat. no. U886060) were purchased from Andy gene (Beijing, China). ROS detection kit (cat. no. S0033) was purchased from Beyotime (Shanghai, China). Lipopolysaccharides (LPS, cat. no. L8880), BCA protein detection kits (cat. no. PC0020), and N‐Acetylcysteine (NAC, cat. no. C8460) were purchased from Solarbio (Beijing, China).

All antibodies in this study were commercially available. Rabbit monoclonal antibodies against NLRP3 (cat. no. 15101) and ABCG2 (cat. no. 4477) were purchased from Cell Signaling Technology (Danvers, MA, USA). Rabbit polyclonal antibodies against Cleaved‐IL‐1*β* (cat. no. WL00891) and TMS1/ASC (cat. no. WL02462) were obtained from Wanleibio (Shenyang, China), and NEK7 (Rabbit pAb, cat. no. DF4467) was sourced from Affinity Biosciences (Jiangsu, China). Rabbit monoclonal antibody against Cleaved‐Caspase 1 (cat. no. A16792) was purchased from ABclonal (Wuhan, China). Mouse monoclonal antibody against *β*‐actin (cat. no. 2D4H5) was purchased from Proteintech (Wuhan, China).

### 2.2 Animal experiment

Male Kunming mice (weighing 22 ± 2 g) were purchased from Liaoning Changsheng Biotechnology Co. Ltd. The mice were allowed to acclimatize for 1 week at 22 ± 2°C with 55% relative humidity.

#### 2.2.1 Establishment and treatment of the mice model of hyperuricemia with acute gout

To establish a model of hyperuricemia with acute gout, mice were intraperitoneally injected with 250 mg/kg of potassium oxonate daily for 12 days. On day 10, monosodium urate (MSU, 0.02 mL of 25 mg/mL) was injected into the right ankle joint of the animal using a micro‐syringe to induce the hyperuricemia with acute gout. As a control, physiological saline was injected either intraperitoneally or into the right ankle joint.

Fifty‐six KM mice were randomly divided into seven groups. These groups included the control, model, low‐dose curcumin (Cur‐L, 50 mg/kg), medium‐dose curcumin (Cur‐M, 100 mg/kg), high‐dose curcumin (Cur‐H, 200 mg/kg), allopurinol (All, 10 mg/kg), and colchicine (Col, 1 mg/kg) groups. Curcumin was dissolved in corn oil and administered via oral gavage (50, 100 or 200 mg/kg, 0.1 mL/20 g body weight) once daily for 12 days to evaluate its effect on hyperuricemia and acute gout. Mice receiving an equal volume of corn oil were used as the blank control, while allopurinol and colchicine served as positive controls.

#### 2.2.2 Measurement of ankle joint swelling

At 0, 4, 12, 24, and 48 h after MSU injection, the circumference of the right ankle of the mice was measured. The percentage of ankle edema was calculated relative to the baseline measurement at 0 h. The presence of a swelling on the opposite side of the joint capsule was considered the criterion for successful injection. Blood and organ samples were collected at 48 h for further analysis.

#### 2.2.3 Xanthine oxidase activity, uric acid content, and renal function assessment

Forty milligrams of mouse liver tissue were homogenized with physiological saline at a 1:9 ratio under ice‐cold conditions using a mechanical homogenizer. XOD activity in the liver was assessed using a commercial kit. Serum levels of UA, BUN and CR were measured.

#### 2.2.4 Measurement of TNF‐*α* and IL‐1*β* inflammatory cytokines

For the ankle joint and kidney tissue samples, the right ankle joint and kidneys of the mice were harvested, and pre‐chilled physiological saline (1:10, g/mL) was added. The tissues were homogenized on ice using a tissue grinder, and then centrifuged at 12,000 rpm for 10 min at 4°C. The supernatant was collected for the measurement of TNF‐*α* and IL‐1*β* levels.

#### 2.2.5 Protein extraction and Western blot analysis

Mouse kidneys were homogenized in the RIPA lysis buffer containing PMSF and aprotinin, and centrifuged at 12,000 rpm for 10 min at 4°C. Equal amount (20 μg) of protein from the supernatants were denatured using SDS sample buffer and separated by SDS‐PAGE. The proteins were then transferred to PVDF membranes, which were incubated overnight with the primary antibodies against ABCG2 or *β*‐actin. The PVDF membranes were incubated with the corresponding HRP‐conjugated secondary antibody for 1 h at 24°C. Protein bands were then visualized using ECL reagent and captured with the ChemiDoc™ Touch Imaging System.

### 2.3 Cell culture

HK‐2 cells and RAW264.7 cells were cultured in DMEM/F12 and DMEM medium with 10% FBS and 1% penicillin/streptomycin.

#### 2.3.1 Establishment and treatment of the model

HK‐2 cells were stimulated with 200 μM MSU for 24 h to establish a cell model for hyperuricemia. RAW264.7 cells were stimulated with 10 μg/mL LPS for 4 h, followed by 200 μM MSU for an additional 20 h to create a cell model for acute gout. The HK‐2 and RAW264.7 cells were divided into control, model, curcumin (40 μM), and NAC (5 mM) groups. Curcumin or the ROS scavenger NAC was added 1 h prior to model induction.

#### 2.3.2 MTT Assay

The cells were seeded in 96‐well plates. After drug treatment, MTT solution was added and incubated for 4 h. The DMSO was added to each well. The absorbance of each well was measured at 490 nm by the microplate reader.

#### 2.3.3 Detection of reactive oxygen species ROS

The DCFH‐DA probe (10 μM) was added and incubated for 20 min. Each well was flushed and observed using a laser scanning confocal microscope.

#### 2.3.4 Protein extraction from cells and protein blot analysis

RAW264.7 or HK‐2 cells were homogenized and centrifuged. The supernatants were collected to assess the expression of NLRP3, NEK7, ASC, Cleaved‐Caspase 1, Cleaved‐IL‐1*β*, GSDMD, ABCG2 or *β*‐actin proteins by Western blotting.

#### 2.3.5 Molecular docking

The crystal structure of the core targets NEK7 and NLRP3 was obtained from the PDB database and imported into AutoDockTools, where the receptor protein dehydration parameters were adjusted and converted into pdbqt files. The 3D structure of curcumin was retrieved from the PubChem database, saved in SDF format, and imported into PyMol for parameter setting, dehydration, and hydrogenation treatment, then converted into pdb format. The three pdbqt files were subsequently imported into AutoDock Tools for molecular docking, and the docking results were evaluated based on binding energy and binding site numbers. Finally, the results were visualized using PyMol software.

#### 2.3.6 Molecular dynamics simulation

To study the structural stability and dynamic interactions of the curcumin‐NEK7‐NLRP3 complex, a 100 ns molecular dynamics simulation was performed using GROMACS. CHARMM36 and GAFF2 were used as the force fields for the protein and ligand, respectively, and the TIP3P water model with periodic boundary conditions was applied. Energy minimization was then carried out using the steepest descent method followed by an isothermal‐isovolumetric ensemble equilibration as well as an isothermal‐isobaric ensemble equilibration system. The simulation was conducted at a constant temperature of 300 K and a pressure of 1 bar for 100 ns.

#### 2.3.7 CETSA

RAW264.7 cells from the gout model were collected using cold PBS containing protease inhibitors and subjected to five cycles of freezing and thawing in liquid nitrogen. The cells were then centrifuged to obtain the cell lysate supernatant. The supernatant was incubated with either DMSO or 100 μM curcumin for 2 h on ice to allow curcumin binding to the proteins. The lysates were subsequently treated at each temperature ranging from 37 to 67°C for 3 min, followed by centrifugation. Western blot analysis was used to detect the levels of NLRP3 and NEK7 proteins bound to curcumin.

### 2.4 Statistical analysis

All data were analyzed using one‐way analysis of variance with GraphPad Prism 9.0.0 to calculate the *p*‐value. Data are presented as mean ± standard error of the mean (SEM). Statistical significance: ns, no significance versus the model group; #*p* < 0.05, ##*p* < 0.01 versus the control group; ∗*p* < 0.05, ∗∗*p* < 0.01 versus the model group.

## 3 RESULTS

### 3.1 Curcumin alleviates inflammation in the mouse model of hyperuricemia with acute gout

**FIGURE 1:**
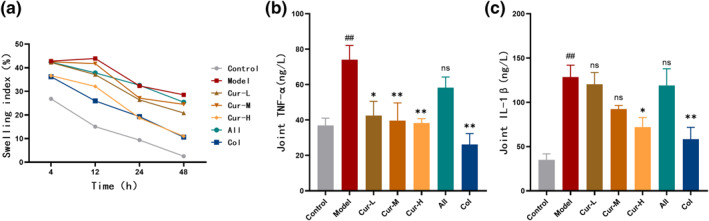
Curcumin alleviates the swelling and inflammation in the ankle joints in the hyperuricemia with acute gout mouse model. (A) Time course of ankle joint swelling (*n* = 8). (B–C) Levels of TNF‐*α* and IL‐1*β* detected in the ankle joint tissue (*n* = 4).

To investigate the anti‐inflammatory effects of curcumin on hyperuricemia‐associated gout, we first evaluated ankle joint swelling in mice. Compared to the control group, the model group exhibited pronounced ankle joint swelling, with the joint swelling at 48 h (28.5 ± 4.1%) significantly higher than that in the control group (2.5 ± 2.3%) (*p* < 0.01). Treatment with curcumin alleviated this swelling, with the high‐dose curcumin group achieving effects comparable to the positive control drug colchicine (Figure 1A). Furthermore, the levels of the TNF‐*α* and IL‐1*β* in the ankle joint tissues were significantly increased in the model group (74.0 ± 8.1 ng/L, *p* < 0.01; 128.6 ± 13.4 ng/L, *p* < 0.01) compared to the control group (36.9 ± 4.2 ng/L, 35.0 ± 6.7 ng/L) (Figure 1B,C). High‐dose curcumin significantly reduced the levels of TNF‐*α* (*p* < 0.01) and IL‐1*β* (*p* < 0.05). These findings indicate that curcumin effectively mitigates ankle joint inflammation in the acute gout model mice.

### 3.2 Curcumin decreases uric acid levels in the mouse model of hyperuricemia with acute gout

Next, we measured the serum uric acid levels in the model mice. Compared to the control group (33.5 ± 2.3 mg/L), the model group exhibited a significant increase in serum uric acid levels (59.7 ± 2.4 mg/L, *p* < 0.01), confirming the successful establishment of the hyperuricemia with acute gout mouse model. High‐dose curcumin treatment significantly reduced UA levels compared to the model group (*p* < 0.01).

XOD is the key enzyme that catalyzes the conversion of xanthine and hypoxanthine to UA. Compared to the control group (4.95 ± 0.37 U/gprot), liver XOD activity was significantly increased in the model group (8.96 ± 0.62 U/gprot, *p* < 0.01). However, XOD activity was significantly reduced in the high‐dose curcumin treatment group (*p* < 0.01), indicating that curcumin may inhibit uric acid production and consequently lower UA levels.

**FIGURE 2:**
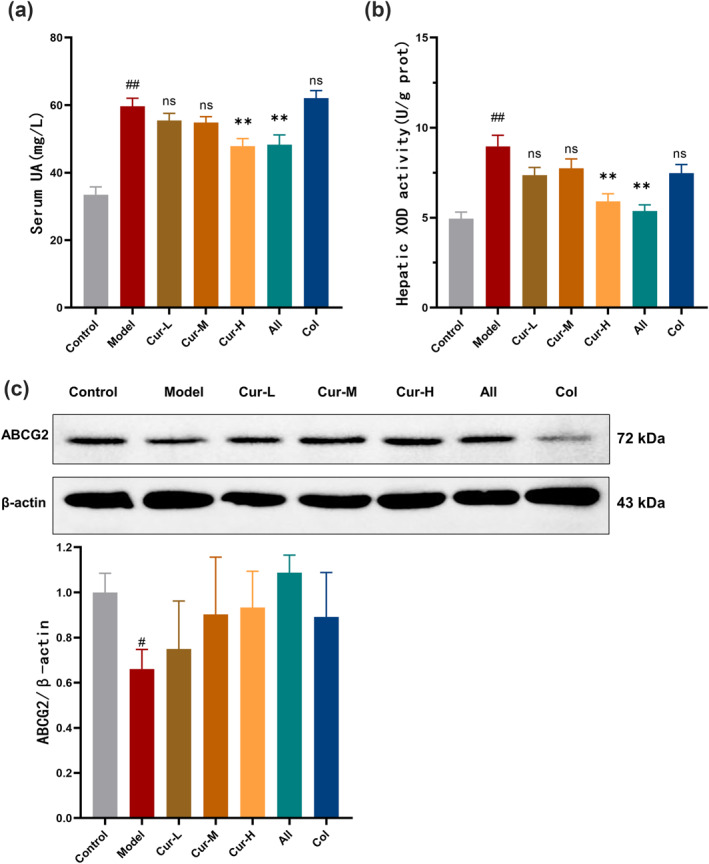
Curcumin reduces serum uric acid accumulation in hyperuricemia with acute gout mouse model. (A) Serum uric acid levels in different groups (*n* = 8). (B) Liver xanthine oxidase activity in different groups (*n* = 8). (C) ABCG2 protein expression in kidney tissues in different groups (*n* = 3).

The regulation of uric acid homeostasis largely relies on the activity of urate transporters in HK‐2 cells. In the model group, renal ABCG2 protein expression was notably decreased compared to the control group, whereas in the curcumin treatment group, ABCG2 protein expression levels were comparable to those of the control group. These findings suggest that curcumin may facilitate uric acid excretion by modulating the expression of urate transporters (Figure 2).

### 3.3 Curcumin mitigates renal dysfunction and inflammation in the mouse model of hyperuricemia with acute gout

**FIGURE 3:**
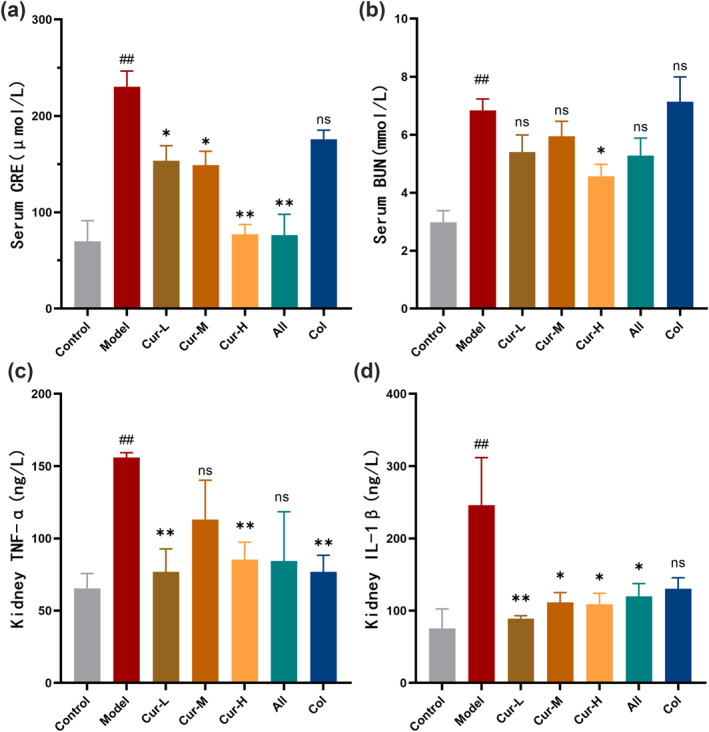
Curcumin mitigates renal dysfunction and inflammation in hyperuricemia in a gout mouse model. Effects of various treatments were detected on serum creatinine levels (A) (*n* = 8), Serum urea nitrogen levels (B) (*n* = 8), Kidney tissue levels of TNF‐*α* (C) (*n* = 3), and Kidney tissue levels of IL‐1*β* (D) (*n* = 3), respectively.

Hyperuricemia is known to contribute to kidney damage. To evaluate the protective effects of curcumin renal injury, we assessed serum levels of creatinine and urea nitrogen. As shown in Figure 3A,B, the model group exhibited significantly elevated serum creatinine (230.3 ± 16.3 μM, *p* < 0.01) and urea nitrogen (6.8 ± 0.4 mM, *p* < 0.01) compared to the control group (70.0 ± 21.2 μM, 3.0 ± 0.4 mM). Treatment with curcumin significantly reduced serum creatinine (*p* < 0.01) and urea nitrogen levels (*p* < 0.05), indicating its potential to mitigate hyperuricemia‐induced kidney damage.

Next, we measured the levels of the pro‐inflammatory cytokines TNF‐*α* (Figure 3C) and IL‐1*β* (Figure 3D) in the kidney tissue of the mice. Compared to the control group (65.4 ± 10.3 ng/L, 75.3 ± 27.3 ng/L), the model group exhibited significantly increased levels of TNF‐*α* (155.9 ± 3.4 ng/L, *p* < 0.01) and IL‐1*β* (245.9 ± 65.9 ng/L, *p* < 0.01). High‐dose curcumin treatment significantly reduced the levels of TNF‐*α* (*p* < 0.01) and IL‐1*β* (*p* < 0.05).

### 3.4 Curcumin inhibits oxidative stress response

**FIGURE 4:**
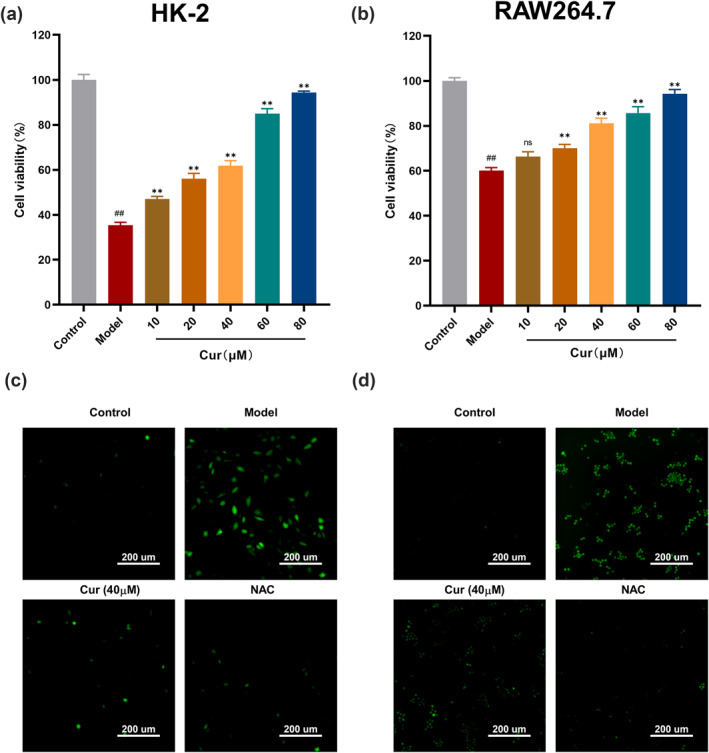
Protective effect of curcumin on oxidative stress. (A) Influencing cell survival on LPS + MSU‐treated RAW264.7 cells (*n* = 6). (B) Influencing cell survival on LPS + MSU‐treated RAW264.7 cells (*n* = 6). (C) Change in reactive oxygen species (ROS) accumulation in HK‐2 cells. (D) Change in ROS accumulation in RAW264.7 cells.

To further explore the mechanism of curcumin in hyperuricemia and acute gout, we established hyperuricemia model in HK‐2 renal tubular epithelial cells and an inflammation gout model in RAW264.7 macrophages. In Figure 4A,B, compared to the control group, cell viability in the model groups was significantly reduced (35.5 ± 1.2%, *p* < 0.01; 60.1 ± 1.3%, *p* < 0.01). However, curcumin, at concentrations ranging from 10 to 80 μM, dose‐dependently inhibited cell death. To explore the effect of curcumin on oxidative stress, we used the ROS scavenger NAC as positive control. In both cell models (Figure 4C,D), the fluorescence intensity of ROS was increased in the model group compared to the control group. Pre‐treatment with NAC dramatically suppressed ROS fluorescence intensity. Similarly, curcumin treatment (40 μM) significantly reduced the ROS levels compared to the model group, effectively inhibiting oxidative stress with effects comparable to NAC (Figure 4C,D).

### 3.5 Curcumin inhibits pyroptosis by modulating the ROS/NEK7‐NLRP3 signaling pathway

**FIGURE 5:**
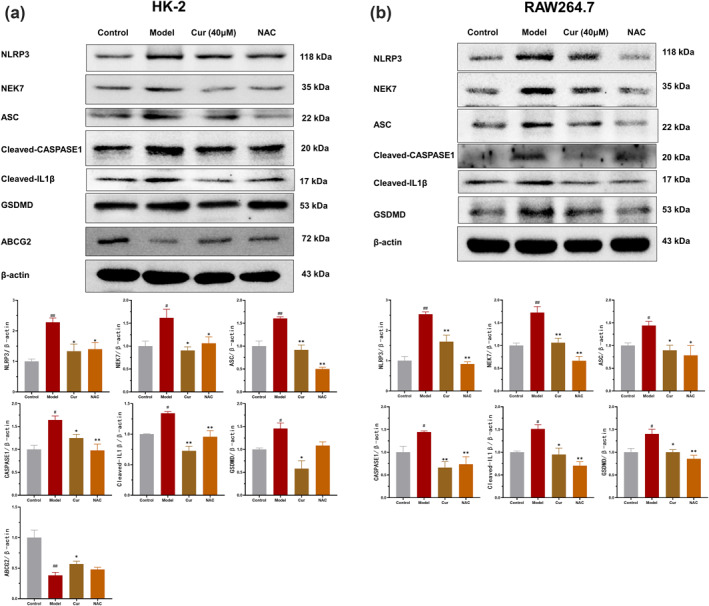
Effect of curcumin on NLRP3‐mediated pyroptosis. (A) Western blot analysis of NLRP3, NEK7, ASC, Cleaved‐CASPASE1, Cleaved‐IL‐1*β*, GSDMD and ABCG2 expression in HK‐2 cells (*n* = 3). (B) Western blot analysis of NLRP3, NEK7, ASC, Cleaved‐CASPASE1, Cleaved‐IL‐1*β* and GSDMD expression in RAW264.7 cells (*n* = 3). The relative expression levels of proteins were quantified using ImageJ software.

Abnormal accumulation of ROS can activate the NLRP3 inflammasome, which plays a key role in pyroptosis. To explore the effects of curcumin on the NLRP3 inflammasome‐mediated pyroptosis pathway, we performed Western blot analysis. The results (Figure 5) revealed that, compared to the control group, the expression levels of NLRP3, NEK7, ASC, Cleaved‐CASPASE1, GSDMD, and Cleaved‐IL‐1*β* were markedly elevated in both cell models, indicating the occurrence of pyroptosis. Treatment with curcumin (40 μM) effectively reversed these changes, producing effects comparable to those of the ROS scavenger NAC.

Furthermore, in the HK‐2 cell model, both curcumin and NAC restored ABCG2 protein expression, providing additional evidence that curcumin promotes UA excretion and lowers serum UA levels in the model mice. These findings suggest that curcumin inhibits the levels of NEK7 and the release of Cleaved‐IL‐1*β*, as well as the activation of CASPASE1 and GSDMD, thereby suppressing pyroptosis in the hyperuricemia and gout inflammation via the ROS/NEK7‐NLRP3 pathway.

### 3.6 Curcumin disrupts the binding between NEK7 and NLRP3

**FIGURE 6:**
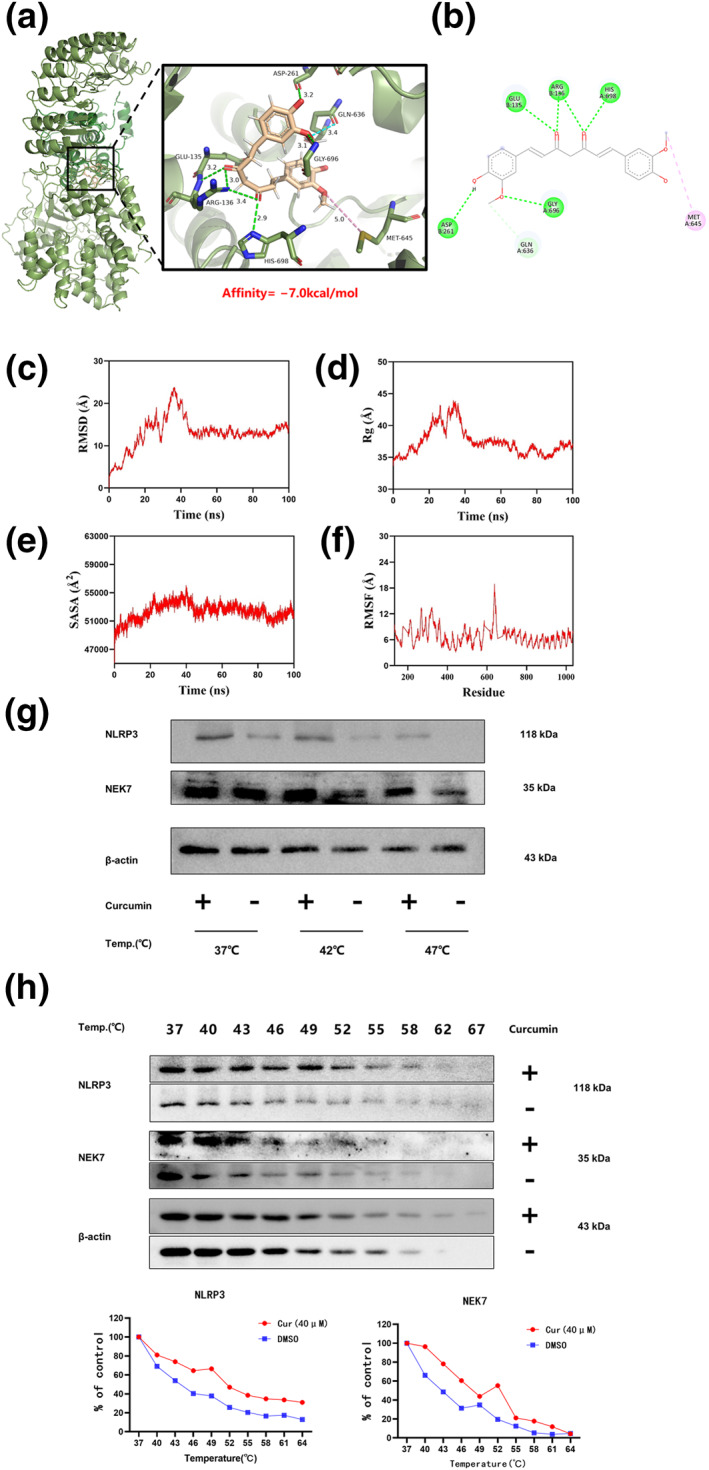
Curcumin disturbs the interaction of NLRP3 and NEK7. (A) Molecular docking simulations for curcumin binding to NLRP3 and NEK7. (B) The targeting sites of curcumin at the docking pocket interface. (C–F) The molecular dynamics simulation results of RMSD (C), Rg (D), SASA (E), and RMSF (F) calculated during 100 ns. (G) Western blot analysis of curcumin binding to NLRP3 and NEK7 in gout model cells at different temperatures (37°C, 45°C, and 53°C) as assessed by CETSA. (H) CETSA‐based Western blot and quantitative analysis of curcumin binding to NLRP3 and NEK7 at various temperatures, represented as a thermal melting curve.

To further investigate the interaction between curcumin and the NLRP3‐NEK7 complex, molecular docking (Figure 6A,B) and molecular dynamics simulations (Figure 6C–F) were conducted. The analysis identified a binding pocket at the interface of NLRP3 and NEK7 where curcumin was found to bind to both proteins with a binding energy of −7.0 kcal/mol.

In addition, based on the principle of protein thermal stability, the Cellular Thermal Shift Assay (CETSA) was employed to evaluate the binding of curcumin to NEK7 and NLRP3 (Figure 6G,H). The CETSA results demonstrated that curcumin binding increased the thermal stability of NEK7 and NLRP3 compared to the drug‐free control group. Specifically, the amount of undigested NEK7 or NLRP3 protein increased the same temperature (Figure 6G,H), and the thermal melt curve of the complex protein showed a rightward shift (Figure 6H). These findings suggest that curcumin may selectively disrupt the interaction between NLRP3 and NEK7, thereby inhibiting the assembly and activation of the NLRP3 inflammasome.

## 4 DISCUSSION

Gout is a prevalent inflammatory arthritis primarily driven by persistent elevation of serum urate levels, which facilitates the formation and deposition of MSU in the joints and other tissues.^[28]^ Traditionally, experimental models of hyperuricemia and acute gout are studied independently in mice.[^29^, ^30^] In this study, we established a combined mouse model of hyperuricemia and acute gout through intraperitoneally injecting potassium oxonate followed by acute administration of MSU. This approach replicates both elevated serum urate levels and ankle joint swelling, providing a more representative model of gout pathology.^[31]^

Curcumin has demonstrated a favorable safety profile in various animal models showing no hepatic, renal, or cardiovascular toxicity.^[23]^ Even in clinical trials where participants consumed up to 8000 mg of curcumin daily for 3 months, no toxic effects were reported.^[24]^

In this study, curcumin administration at a dose of 200 mg/kg significantly inhibited hepatic xanthine oxidase activity in the model mice, resulting in reduced serum levels of urate, creatinine, and blood urea nitrogen as well as alleviation of joint swelling. Additionally, curcumin notably increased the expression of urate transporter ABCG2 in the kidneys, suggesting its potential to ameliorate hyperuricemia and gout symptoms.

ABCG2, localized on the basolateral membrane, plays a pivotal role in maintaining the balance between urate secretion and reabsorption and is closely linked to the pathogenesis of hyperuricemia.^[32]^ Damage to renal tubular epithelial cells is a critical factor in urate‐induced kidney injury.^[33]^ Studies have demonstrated that IL‐1*β* can suppress ABCG2 expression by downregulating the PDZK1 protein.^[34]^ Therefore, the regulatory mechanism of curcumin on ABCG2 protein expression may involve the inhibition of IL‐1*β* release, thereby promoting the activation of ABCG2.

In this study, the model group exhibited elevated IL‐1*β* levels and significantly suppressed ABCG2 expression, indicative of the impaired urate excretion. However, curcumin treatment reversed these effects by increasing ABCG2 protein expression, which likely facilitated renal urate excretion. These findings align with previous studies demonstrating the anti‐inflammatory properties of curcumin in alleviating renal inflammation.

The study demonstrated that curcumin treatment significantly reduced intracellular ROS levels induced by LPS and/or MSU in cultured cells as well as the protein levels of NLRP3, NEK7, ASC, cleaved‐CASPASE1, cleaved‐IL‐1*β* and GSDMD, thereby inhibiting pyroptosis mediated through the NLRP3 inflammasome signaling pathway. NEK7, one of the 11 identified NEK kinases, is ubiquitously expressed in eukaryotic cells and plays a critical role in mitosis. Furthermore, NEK7 serves as a key regulator of potassium efflux and is considered an upstream mediator of NLRP3 inflammasome activation. Upon stimulation by danger signals such as MSU crystals, amyloid proteins and ATP, NEK7 regulates activation of NLRP3 inflammasomes.

To elucidate the mechanism underlying curcumin's inhibitory effect on NLRP3 inflammasome activation, molecular docking simulations were employed. These simulations predicted binding interactions of curcumin with NEK7 and NLRP3. Additionally, CETSA, which relies on the principle that protein stability increases upon drug binding, serves as a robust method for evaluating drug‐target protein interactions. In this study, CETSA was used to validate the binding of curcumin to NEK7 and NLRP3. CETSA results showed minimal degradation of the curcumin‐protein complexes at various temperatures. The findings indicate that curcumin may inhibit the assembly and activation of the NLRP3 inflammasome by directly targeting and disrupting the interaction between NEK7 and NLRP3, thus providing insight into curcumin's ability to mitigate gout‐related damage and inflammation.

Curcumin, a molecule exhibiting diverse pharmacological effects, possesses multiple modifiable functional groups within its structural framework. Recent studies have demonstrated that structural modifications to this core scaffold—particularly through the design of curcumin derivatives and copper (II) complexes—enable these compounds with stimuli‐responsive properties.^[35]^ This suggests that curcumin can be a promising multi‐targeting molecular scaffold for rational drug design, especially in the structural optimization to improve its efficacy, targeting specificity, and pharmacokinetic profile. Further research is needed to elucidate the mechanisms underlying curcumin's smart interactions with biological targets.

## CONFLICT OF INTEREST STATEMENT

The authors declare no conflicts of interest.

## ETHICS STATEMENT

All animal experiments were approved by the Biomedical Ethics Committee of Dalian University of Technology.

## Data Availability

All data generated or analyzed during this study are included in this published article.
